# Tracking of Antibiotic Resistance Transfer and Rapid Plasmid Evolution in a Hospital Setting by Nanopore Sequencing

**DOI:** 10.1128/mSphere.00525-20

**Published:** 2020-08-19

**Authors:** Silke Peter, Mattia Bosio, Caspar Gross, Daniela Bezdan, Javier Gutierrez, Philipp Oberhettinger, Jan Liese, Wichard Vogel, Daniela Dörfel, Lennard Berger, Matthias Marschal, Matthias Willmann, Ivo Gut, Marta Gut, Ingo Autenrieth, Stephan Ossowski

**Affiliations:** a Institute of Medical Microbiology and Hygiene, University of Tübingen, Tübingen, Germany; b German Center for Infection Research (DZIF), Partner Site Tübingen, Tübingen, Germany; c Centre for Genomic Regulation (CRG), The Barcelona Institute of Science and Technology, Barcelona, Spain; d Institute of Medical Genetics and Applied Genomics, University of Tübingen, Tübingen, Germany; e CNAG-CRG, Centre for Genomic Regulation (CRG), Barcelona Institute of Science and Technology (BIST), Barcelona, Spain; f Medical Center, Department of Hematology, Oncology, Immunology, Rheumatology & Pulmonology, University of Tübingen, Tübingen, Germany; g Clinical Collaboration Unit Translational Immunology, German Cancer Consortium (DKTK) and German Cancer Research Center (DKFZ), Partner Site Tübingen, Tübingen, Germany; h Universitat Pompeu Fabra (UPF), Barcelona, Spain; i Barcelona Supercomputing Center, BSC, Barcelona, Spain; Antimicrobial Development Specialists, LLC

**Keywords:** plasmids, Nanopore, long read, IMP-8, *Pseudomonas aeruginosa*, *pathoLogic*, *plasmIDent*, genome assembly, horizontal gene transfer, Nanopore sequencing, antimicrobial resistance, plasmid-mediated resistance, surveillance studies

## Abstract

Infections with multidrug-resistant bacteria often leave limited or no treatment options. The transfer of antimicrobial resistance genes (ARG) carrying plasmids between bacterial species by horizontal gene transfer represents an important mode of expansion of ARGs. Here, we demonstrate the application of Nanopore sequencing in a hospital setting for monitoring transfer and rapid evolution of antibiotic resistance plasmids within and across multiple species. In 2009, we experienced an outbreak with extensively multidrug-resistant Pseudomonas aeruginosa harboring the carbapenemase-encoding *bla*_IMP-8_ gene. In 2012, the first Citrobacter freundii and Citrobacter cronae strains harboring the same gene were detected. Using Nanopore and Illumina sequencing, we conducted comparative analysis of all *bla*_IMP-8_ bacteria isolated in our hospital over a 6-year period (*n* = 54). We developed the computational platform *plasmIDent* for Nanopore-based characterization of clinical isolates and monitoring of ARG transfer, comprising *de novo* assembly of genomes and plasmids, plasmid circularization, ARG annotation, comparative genome analysis of multiple isolates, and visualization of results. Using *plasmIDent*, we identified a 40-kb plasmid carrying *bla*_IMP-8_ in P. aeruginosa and C. freundii, verifying the plasmid transfer. Within C. freundii, the plasmid underwent further evolution and plasmid fusion, resulting in a 164-kb megaplasmid, which was transferred to C. cronae. Multiple rearrangements of the multidrug resistance gene cassette were detected in P. aeruginosa, including deletions and translocations of complete ARGs. In summary, plasmid transfer, plasmid fusion, and rearrangement of the ARG cassette mediated the rapid evolution of opportunistic pathogens in our hospital. We demonstrated the feasibility of near-real-time monitoring of plasmid evolution and ARG transfer in clinical settings, enabling successful countermeasures to contain plasmid-mediated outbreaks.

**IMPORTANCE** Infections with multidrug-resistant bacteria represent a major threat to global health. While the spread of multidrug-resistant bacterial clones is frequently studied in the hospital setting, surveillance of the transfer of mobile genetic elements between different bacterial species was difficult until recent advances in sequencing technologies. Nanopore sequencing technology was applied to track antimicrobial gene transfer in a long-term outbreak of multidrug-resistant Pseudomonas aeruginosa, Citrobacter freundii, and Citrobacter cronae in a German hospital over 6 years. We developed a novel computational pipeline, *pathoLogic*, which enables *de novo* assembly of genomes and plasmids, antimicrobial resistance gene annotation and visualization, and comparative analysis. Applying this approach, we detected plasmid transfer between different bacterial species as well as plasmid fusion and frequent rearrangements of the antimicrobial resistance gene cassette. This study demonstrated the feasibility of near-real-time tracking of plasmid-based antimicrobial resistance gene transfer in hospitals, enabling countermeasures to contain plasmid-mediated outbreaks.

## Abstract (precis)

Infections with multidrug-resistant bacteria represent a major threat to global health. While the spread of multidrug-resistant bacterial clones is frequently studied in the hospital setting, surveillance of the transfer of mobile genetic elements between different bacterial species was difficult until recent advances in sequencing technologies. Nanopore sequencing technology was applied to track antimicrobial gene transfer in a long-term outbreak of multidrug-resistant Pseudomonas aeruginosa, Citrobacter freundii, and Citrobacter cronae in a German hospital over 6 years. We developed a novel computational pipeline, *pathoLogic*, which enables *de novo* assembly of genomes and plasmids, antimicrobial resistance gene annotation and visualization, and comparative analysis. Applying this approach, we detected plasmid transfer between different bacterial species as well as plasmid fusion and frequent rearrangements of the antimicrobial resistance gene cassette. This study demonstrated the feasibility of near-real-time tracking of plasmid-based antimicrobial resistance gene transfer in hospitals, enabling countermeasures to contain plasmid-mediated outbreaks.

## INTRODUCTION

The increase in the number of multidrug-resistant (MDR) bacterial strains has led organizations such as the World Health organization (WHO) and the U.S. Centers for Disease Control and Prevention (CDC) to categorize MDR bacteria as representing a major public health problem (1). Infection of patients with MDR bacteria often leaves only very limited or even no treatment options, thus posing a potentially life-threatening risk to individual patients, in particular, those in intensive care units (2, 3). In addition, infection control measures to prevent spreading are required, resulting in increased efforts with respect to patient care and increased costs for health care providers and public health care systems (4, 5). Although action is needed on different national and international levels, understanding colonization, infection, and transmission routes of these MDR-resistant bacteria in the local hospital setting represents a crucial initial step toward implementation of harmonized, successful strategies to combat infections caused by MDR bacteria (1, 4).

Next-generation sequencing (NGS) has become widely available and has been used successfully to resolve outbreaks and determine transmission routes (see, e.g., the review in reference 6). However, both the clonal transmission of MDR bacteria and the spread of multidrug resistance by horizontal gene transfer (HGT) between different bacterial species represent important modes of expansion of antimicrobial resistance (AMR) genes (7). Although multidrug resistance plasmids and plasmid transfer have been studied in hospital settings, their interrogation is not part of routine infection control practice. Moreover, methods of plasmid characterization and comparisons based on short-read sequences are error prone and unreliable, particularly when larger (>50-kb) plasmids are involved (8), while long-read *de novo* assembly-based plasmid analysis is currently limited to large centers with access to Pacific Biosystems (PacBio) Sequel sequencers (see, e.g., references 9 and 10). Recently, the MinION long-read sequencer (Oxford Nanopore Technologies [ONT]) became more widely available, facilitating fast and inexpensive analysis of multidrug resistance plasmids and horizontal gene transfer and evolution of plasmid-born antimicrobial resistance (AMR) (11, 12). Thus, the technology is potentially suitable for application within the hospital setting. In recent publications, Dong et al. examined the microevolution of *bla*_KPC_ harboring plasmids in three clinical isolates applying Nanopore technology (13), while Lemon et al. optimized the Nanopore sequencing laboratory workflow and analyzed plasmids from three clinical isolates (11). Long-read sequences substantially increase the contiguity of *de novo* assemblies by spanning repeat regions, resulting in finished microbial genome and plasmid assemblies (14). However, due to the high error rates of Nanopore sequencing, hybrid assemblers such as hybridSPAdes (15) and Unicycler (16) combine long and short reads to achieve a high base level of the accuracy needed for the correct identification of AMR-related genes and variants. In the present study, we aimed to evaluate the application of Nanopore sequencing technology in a hospital setting and to demonstrate the feasibility of monitoring transfer and rapid evolution of antibiotic resistance plasmids within and across multiple species.

Starting in 2009, our hospital experienced an outbreak caused by an extensively multidrug-resistant Pseudomonas aeruginosa clone (17). The strain harbored a carbapenemase-encoding gene (*bla*_IMP-8_), which renders most beta-lactams ineffective, including carbapenems, an antibiotics class of last resort (18). Extensive infectious disease interventions and the establishment of a rectal screening program to identify colonized patients led to a reduction of cases. However, in March 2012, we detected the first Citrobacter freundii strain harboring the same carbapenemase-encoding *bla*_IMP-8_ gene (19), approximately 2.5 years after the first P. aeruginosa *bla*_IMP-8_ gene had been detected. Shortly after, the carbapenemase was detected in Citrobacter cronae (20). Since *bla*_IMP-8_ is rarely encountered in Europe and Germany (21, 22) and has not yet been detected in rectal screening swabs from patients submitted to our hospital for the first time, we hypothesized that horizontal gene transfer had occurred within bacterial strains circulating in our hospital. Therefore, we conducted a sequencing study that included all multidrug-resistant bacteria harboring the *bla*_IMP-8_ gene isolated in our hospital over a 6-year period, including patient and environmental isolates. We developed and established a bioinformatics pipeline in order (i) to determine the sequence of the *bla*_IMP-8_-harboring plasmids and characterize all of the AMR genes contained, (ii) to identify potential events of transmission of the plasmids between species, and (iii) to characterize the evolutionary dynamics of the plasmids.

## RESULTS

### Comprehensive analysis platform for antibiotic resistance gene-carrying plasmids.

**FIG 1:**
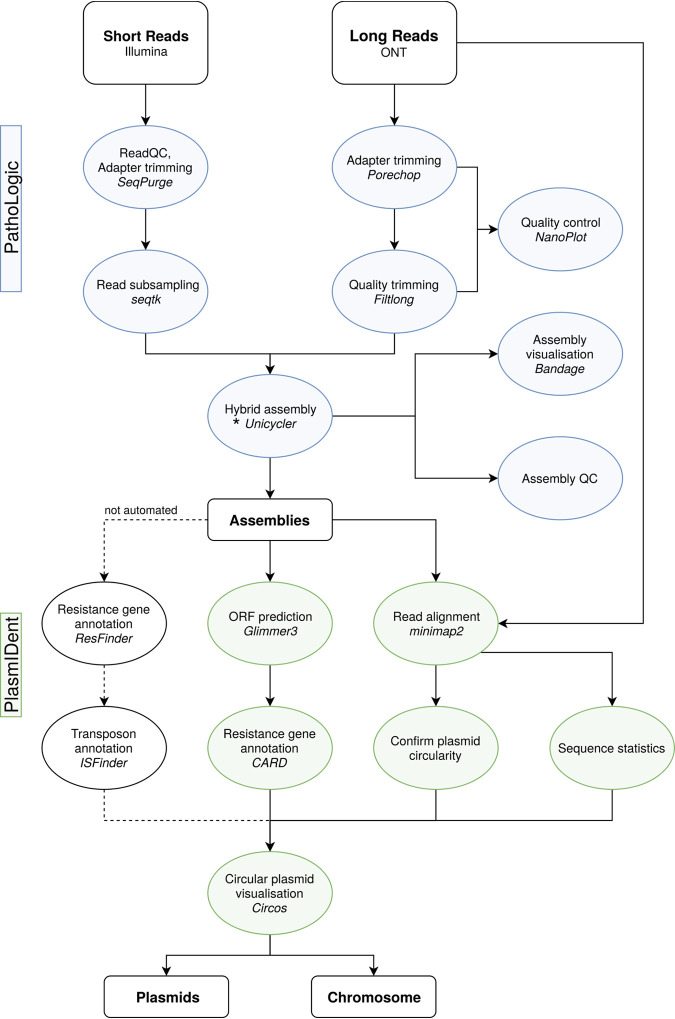
Schematic diagram of the data analysis workflow used in this study. The *pathoLogic* platform was created using the *Nextflow* (39) environment to chain different tools and scripts, represented here as circular nodes. Connecting lines indicate data flow between the separate processes; dashed lines show tools that are not directly included in the pipeline and need manual data handling. In *pathoLogic*, the assembly step (*) can be performed by *Unicycler* (16), *Canu* (40), *miniasm* (41), *hybridSPAdes* (15), or *flye* (42, 43). ORF, open reading frame.

We have developed a comprehensive computational platform for the genomic analysis of clinical isolates and the monitoring of antibiotic resistance gene transfer. *pathoLogic* comprises a hybrid *de novo* assembly pipeline generating finished genomes and plasmids and performing genome polishing, quality control (QC), annotation, and comparative genome analysis of multiple isolates, as well as visualization of results (Fig. 1). Furthermore, *pathoLogic* integrates the *plasmIDent* method, which confirms the circularity of putative plasmids by ring closure using long reads, performs AMR gene annotation, calculates various sequence properties (e.g., GC content and GC skew and coverage depth), and creates a circular visualization of the annotated plasmid. Finally, sequences of plasmids from multiple isolates of the same or different species are compared in order to identify horizontal gene transfers, structural variations (e.g., AMR gene presence/absence), and point mutations, which can further be utilized for phylogenetic or transmission analysis. *pathoLogic*, *plasmIDent* and a graphical user interface (GUI) are freely available on github (*plasmIDent* pipeline, https://github.com/imgag/plasmIDent; *pathoLogic* pipeline, https://github.com/imgag/pathoLogic).

### Characterization of study isolates.

In our study, we included all *bla*_IMP-8_ AMR gene-positive strains isolated in our hospital from patients or patient-related environmental water sources in the hemato-oncology department over a period of 6 years (*n* = 54). This also comprised the previously reported P. aeruginosa outbreak clones (*n* = 34) (17) and one C. freundii *bla*_IMP-8_ isolate (19), for which Illumina short-read data are available (https://www.ebi.ac.uk/ena/browser/view/PRJEB31907). In order to obtain finished genomes and circularized plasmids, long-read Nanopore sequencing was conducted with all Citrobacter freundii (*n* = 8) and Citrobacter cronae (*n* = 1) isolates and selected P. aeruginosa (*n* = 5) isolates representing different time points (see Table S1 in the supplemental material). Applying the *pathoLogic* pipeline described above enabled us to generate high-quality genomes for all samples. We were able to generate a single circular chromosome along with several circular plasmids in 5 of the 14 samples (Table S2). All other assemblies also had a few large contigs, as indicated by a high NG75 value. Samples with a lower depth of coverage of Nanopore reads (e.g., isolate 9_E_CF) also resulted in more-fragmented assemblies.

10.1128/mSphere.00525-20.4 TABLE S1 Quality parameters for ONT data obtained in this study, calculated using *Nanoplot* after adapter trimming performed with *Porechop* (https://github.com/rrwick/Porechop). Also shown are the Flow cell and Reagent kit version used for the Nanopore-sequencing run. Download Table S1, PDF file, 0.2 MB. Copyright © 2020 Peter et al. 2020 Peter et al. This content is distributed under the terms of the Creative Commons Attribution 4.0 International license.

10.1128/mSphere.00525-20.5 TABLE S2 Assembly quality parameters calculated with *QUAST* (44). Download Table S2, PDF file, 0.2 MB. Copyright © 2020 Peter et al. 2020 Peter et al. This content is distributed under the terms of the Creative Commons Attribution 4.0 International license.

### Plasmid content and phylogeny of the study isolates.

**FIG 2:**
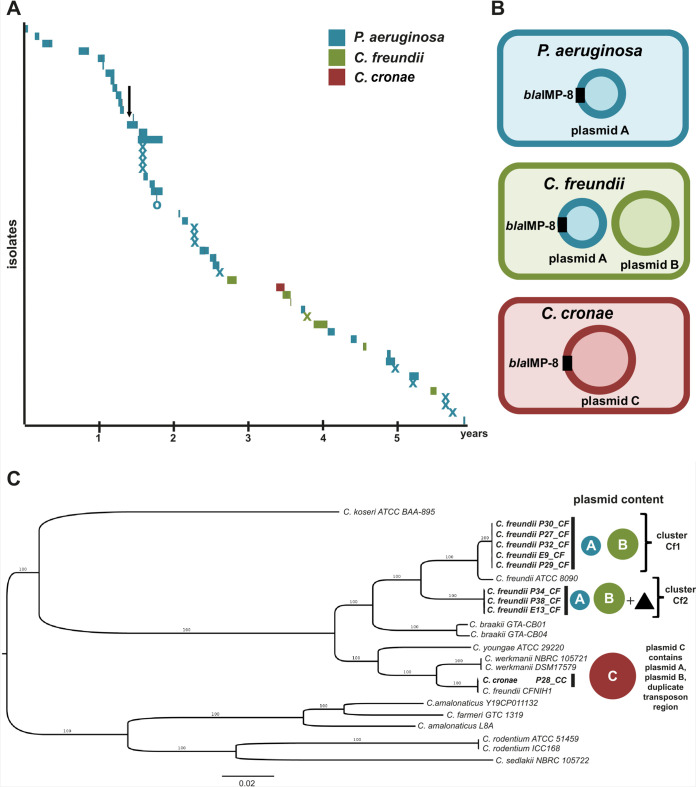
(A) Timeline of isolation of *bla*_IMP-8_ Gram-negative bacteria in the hemato-oncology department over 6 years. Bars represent isolates from patients and the length of their stay in the hospital. Patient 21 was seen only in the outpatient department, marked with an “O.” Environmental isolates are marked with an “X” at the date of isolation. The introduction of a rectal screening program is marked with a black arrow. (B) Overview of plasmids with relevance to the evolution of the *bla*_IMP-8_ plasmid found in P. aeruginosa, C. freundii, and C. cronae. (C) Maximum likelihood phylogeny of *Citrobacter* species included in the study (*n* = 9). The Citrobacter freundii strains formed two clusters, Cf1 (*n* = 5) and Cf2 (*n* = 3). Strains of cluster Cf2 harbored a chromosomal transposon region (black triangle) homologous to the regions of plasmid C. C. cronae clustered with the closely related C. werkmanii NBRC105721 and DSM17579 strains. The scale bar shows the expected number of nucleotide changes per site. PA, P. aeruginosa; CF, Citrobacter freundii; CC, *Citrobacter cronae*.

For the first 2.5 years, we observed *bla*_IMP-8_ in P. aeruginosa isolates from only 26 patients before we first detected C. freundii and C. cronae carrying *bla*_IMP-8_ (Fig. 2A). The plasmids with relevance to the dynamics of the *bla*_IMP-8_ plasmid evolution are displayed in Fig. 2B. The complete plasmid content of all isolates is summarized in Table S3.

10.1128/mSphere.00525-20.6 TABLE S3 Overview of all plasmids identified in the samples. Panel A shows all samples and their respective plasmids, assembled using the hybrid Nanopore/Illumina assembly tool UniCycler. Panel B shows detailed information about all identified plasmids. Plasmids A, B, and C are involved in the resistance gene transmission and plasmid fusion and are discussed in detail in the main manuscript. Plasmids originating from the same ancestor are labeled with the same letter and apostrophes indicating versions with minor variations. Download Table S3, PDF file, 0.3 MB. Copyright © 2020 Peter et al. 2020 Peter et al. This content is distributed under the terms of the Creative Commons Attribution 4.0 International license.

In P. aeruginosa isolates, we detected a 40-kb plasmid carrying the *bla*_IMP-8_ gene (plasmid A, blue). In C. freundii isolates, *bla*_IMP-8_ plasmid A was found in addition to an 88-kb plasmid (plasmid B, green) without a carbapenemase-encoding gene. Surprisingly, in the C. cronae isolate, a large 164-kb plasmid harboring the *bla*_IMP-8_ gene was detected (plasmid C, red) without any evidence of the presence of plasmid A or plasmid B. The structures and circular nature of the three plasmids were confirmed by remapping the long-read sequences, resulting in continuous read coverage along the plasmids without breakpoints.

Phylogenetic analysis showed that all of the P. aeruginosa strains were closely related and belonged to a single cluster, indicating clonal spread (data not shown). All isolated P. aeruginosa strains were found to belong to sequence type 308 (ST308). In contrast, the maximum likelihood phylogeny of the *Citrobacter* isolates revealed a phylogenetically more diverse picture (Fig. 2C). The C. freundii isolates formed two clusters, Cf1 (*n* = 5) and Cf2 (*n* = 3), which were clearly distinct (Fig. 2C). Both clusters contained plasmids A and B. Isolates of cluster Cf2 contained an additional plasmid G (Table S3) and a region containing parts of the Tn*3* family transposons localized on the chromosome absent in cluster Cf1, which is further described below.

### Comparative genomic analysis and annotation of plasmids.

**FIG 3:**
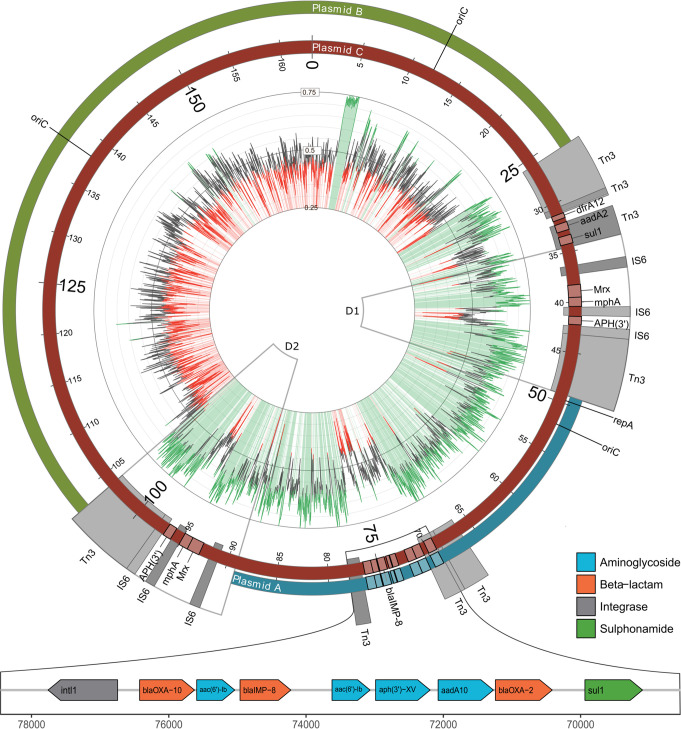
Detailed alignment of plasmids A, B, and C. Plasmid A (blue, outer circle) harbored a multidrug resistance cassette that included *bla_I_*_MP-8_ on a class 1 integron and shows high GC content (inner circle, green). Plasmid B (green, outer circle) harbored no *bla_I_*_MP-8_ resistance gene and shows lower GC content (red). Plasmid C (red) was the largest and comprised plasmid A, plasmid B, duplicated regions 1 and 2 (black arrows), and a unique extension by two Tn*3* elements in one fusion region. Highlighted in blue are hits in the IS finder database annotated to the transposon or IS family level. Parts of Tn*3* family transposons are present in different locations of the plasmids. The class 1 integron consists of 9 AMR genes, including those encoding aminoglycosides, beta-lactams, and sulfonamides. Additional AMR genes and a mercury resistance operon (23) are present within the duplicated region. The resistance gene translocations are displayed in a schematic manner.

Next, we performed multiple-sequence alignment of the generated reference sequences of plasmids A, B, and C (Fig. 3). To better understand the chronological order of the horizontal gene transfer (HGT) and fusion events, we first performed an in-depth annotation of plasmid features, including antimicrobial resistance genes, transposons, origin of replication, and GC content (Fig. 3). Plasmid A, which contains the *bla*_IMP-8_ gene, had average GC content of 59% (Fig. 3, green inner circle). The *bla*_IMP-8_ gene was located on a class 1 integron together with eight additional antimicrobial resistance genes (Fig. 3, bottom). The integron comprised the *intI1* integrase gene and AMR genes *bla*_OXA-10_, *aac(6)-lb*, *bla*_IMP-8_, *qacH*, *aph(3′)-XV*, *aadA10*, *bla*_OXA-2_, and *sul*. Plasmid B had a size of approximately 88 kb and substantially lower (<50%) GC content than plasmid A and lacked the *bla_I_*_MP-8_ integron. The largest plasmid, plasmid C, with a size of 164 kb, was composed of the entirety of plasmid A, including the class I integron harboring the AMR genes, and plasmid B, as well as two large stretches containing the duplicated regions D1 and D2 (Fig. 3). Therefore, plasmid C most likely resulted from a fusion of plasmids A and B. The two duplicated regions between plasmids A and B harbored a duplicated region (marked in Fig. 3 with a black arrow) composed of parts of Tn*3* family transposons, three IS*6* family elements, and several AMR genes. Two additional regions containing parts of transposons of the Tn*3* family interspersed with additional AMR genes extended one of the fusion regions.

Results of a similarity search for all identified plasmids using NCBI Microbial Nucleotide BLAST are shown in Table S3A. Notably, we found plasmid SDENCHOLpb, which is highly similar (97% identity) to 63% of the sequence of plasmid A. However, plasmid SDENCHOLpb, which was isolated from Sterolibacterium denitrificans, lacks the resistance gene cassette found in plasmid A (see Fig. S3 in the supplemental material). SDENCHOLpb was sampled in close geographical proximity to our hospital (the distance from Freiburg, Germany, to Tübingen, Germany, is around 120 km).

### Plasmid content of isolates and plasmid fusion.

**FIG 4:**
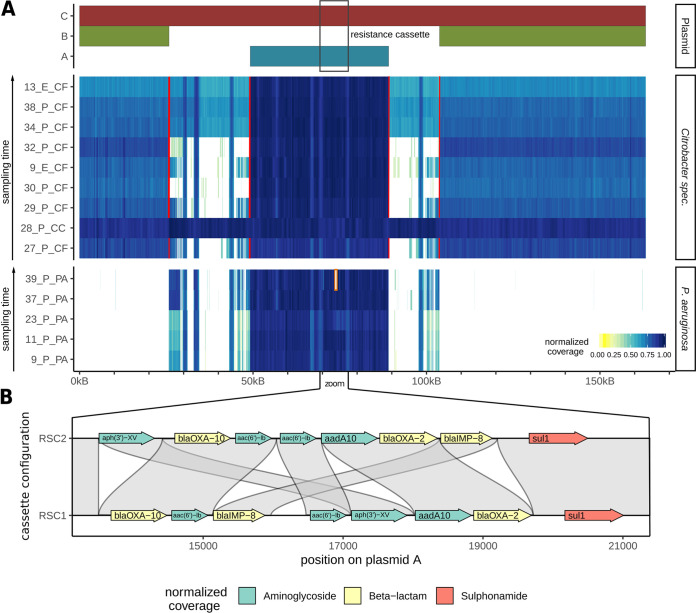
(A) Coverage plot based on short-read Illumina data mapped against reference plasmid C found in C. cronae. Plasmid C (red bar) comprises of the sequences of plasmids A (blue bar) and B (green bar) fused by two transposon-rich regions. Red lines indicate breakpoints, which are characterized by the absence of reads spanning the breakpoint. White areas indicate the absence of coverage and hence the absence of sequence in a given isolate, which in some cases could have been the result of a deletion event or might indicate boundaries between scaffolds. (B) Comparison of the resistance gene cassette configurations of RSC1 and RSC2, showing putative AMR gene translocation events and a deletion of *qacH*.

In order to determine the plasmid content of all studied isolates, we realigned the Illumina short-read sequences using as a reference assembled plasmid C, which comprises the sequences of plasmids A and B and the duplicated regions D1 and D2 (Fig. 3). The coverage for each strain is displayed in Fig. 4. All P. aeruginosa isolates contained only plasmid A and not plasmid B or C. Sequencing reads of P. aeruginosa that mapped to a small section of the transposon-containing region most likely originated from the chromosome. The picture is more complex for the *Citrobacter* species, which could be divided into three groups. The C. cronae (28_P_CC) strain contained the complete C plasmid, which was homogenously covered. The C. freundii isolates formed two groups, one group with plasmid A and B and the second group containing plasmid A and B as well as coverage of the transposon-containing regions. The two groups were found to be identical, with clusters Cf1 and Cf2 distinguished by phylogenetic analysis of the chromosomes.

We further investigated the read coverage distribution for the C. freundii isolates to determine if they harbor only copies of plasmids A and B or instead harbor a combination of copies of plasmids A, B, and C. No continuous short or long reads could be detected spanning the breakpoints between the plasmid A sequence and the plasmid B sequence and duplicated regions D1 and D2 in either of the two C. freundii clusters (Fig. 4; red lines indicate the breakpoints), suggesting that the short reads mapping between A and B originated from a chromosomal integration of the transposon-containing regions. Annotation of the assembled chromosomes of C. freundii and C. cronae isolates confirmed that cluster Cf2 contained the transposon sequence within the chromosomal scaffold whereas cluster Cf1 and C. cronae did not (Fig. S1). We conclude that both Cf1 and Cf2 harbor copies only of plasmids A and B, but not of plasmid C, and that Cf2 harbors a copy of the transposon-containing region in the chromosome.

10.1128/mSphere.00525-20.1 FIG S1 Dot plot visualization of plasmid C (*y* axis) aligned to the complete genomes and plasmids of four representative isolates (*x* axis). Regions without alignments are hidden. The alignment of C. freundii isolate 34_P_CF (Cf2) shows that parts of plasmid C match a region on the chromosome at ca. 850 kbp. This indicates that the Tn*3* regions fusing plasmid A and B to C likely originated in the chromosomes of Citrobacter freundii cluster Cf2. Download FIG S1, TIF file, 0.1 MB. Copyright © 2020 Peter et al. 2020 Peter et al. This content is distributed under the terms of the Creative Commons Attribution 4.0 International license.

Only isolates of cluster Cf2 show a complete “smear” in the coverage plot across the whole transposon-containing region (13_E_CF, 34_P_CF, and 38_P_CF). In isolates of cluster Cf1, however, we observed only partial coverage of the transposon-containing region (for Cf1.1, 3 isolates, including 9_E_CF, 29_P_CF, and 27_P_CF) or almost no coverage (for Cf1.2, 2 isolates, including 32_P_CF and 30_P_CF). Interestingly, the regions distinguishing the Cf1.1 and Cf1.2 subclasses harbor a mercury resistance operon (23) present in Cf1.1 but absent in Cf1.2. Pairwise alignment to the full genomes using the *nucmer* aligner confirmed that these genes are located on plasmid J (Table S3) in isolate 29_P_CF and on noncircular contigs in the other two isolates (9_E_CF and 27_P_CF) of group Cf1.1 (Fig. S1).

In summary, our phylogenetic analysis as well as the comprehensive plasmid annotations indicated that the C. freundii isolates in the Cf1 and Cf2 clusters represent different clones with a mean core single nucleotide polymorphism (SNP) distance of 41,825 nucleotides (minimum, 41,819; maximum, 41,836) and should be treated as separate entities in the identification of plasmid-born horizontal gene transfers.

### Deletion and transposition of AMR genes in P. aeruginosa.

While the P. aeruginosa isolates homogeneously contained only plasmid A, we observed that the resistance gene cassette for some isolates was not continuously covered with short reads in the reference alignment shown in Fig. 4 (see also Fig. S2). Using short-read-based and long-read-based structural variant detection methods, we identified two types of rearrangement events. First, we found various deletions of resistance genes within the resistance gene cassette in 12 strains, indicated by zero coverage (Fig. S2, white areas flanked by red brackets). Analysis of the resistance genes annotated by ResFinder or CARD on the respective plasmid scaffolds confirmed that these deletions correspond to missing AMR genes in the respective strains (Table S4). Furthermore, all deletions were found to span the sequence from exactly the 5′ end to the 3′ end, consisting of one AMR gene plus the flanking *IS* element.

10.1128/mSphere.00525-20.2 FIG S2 Coverage plot of short-read Illumina data mapped against plasmid C. Presented here are only those P. aeruginosa strains for which no Nanopore sequencing data were available. The selected region focuses on the resistance cassette (the reference is cassette configuration RSC1), highlighting a high number of deletions and coverage variations between the different samples. Manually confirmed borders of deleted regions (Fig. S2) are included as red brackets (see also Fig. 4 for coverage plots of samples for which Nanopore data were available). Download FIG S2, TIF file, 0.4 MB. Copyright © 2020 Peter et al. 2020 Peter et al. This content is distributed under the terms of the Creative Commons Attribution 4.0 International license.

10.1128/mSphere.00525-20.3 FIG S3 Pairwise comparison of resistance plasmid A with the published plasmid SDENCHOLpb (GenBank accession no. LT837805.1) reveals highly similar sequences. Plasmid A contains the complete sequence of plasmid SDENCHOLpb, representing around 60% of the sequence of plasmid A. However, SDENCHOLpb does not contain the AMR gene cassette found in plasmid A. The SDENCHOLpb plasmid was sampled in close geographical proximity (the distance from Freiburg, Germany, to Tübingen, Germany, is ∼120km). The SDCNCHOLpb plasmid was isolated from Sterolibacterium denitrificans. Download FIG S3, TIF file, 0.2 MB. Copyright © 2020 Peter et al. 2020 Peter et al. This content is distributed under the terms of the Creative Commons Attribution 4.0 International license.

10.1128/mSphere.00525-20.7 TABLE S4 List of samples with deletions affecting one or more resistance genes. Download Table S4, PDF file, 0.2 MB. Copyright © 2020 Peter et al. 2020 Peter et al. This content is distributed under the terms of the Creative Commons Attribution 4.0 International license.

Moreover, comparing the resistance gene cassettes of P. aeruginosa isolates 37_P_PA and 39_P_PA, we detected breakpoints between AMR genes without a corresponding drop of coverage, indicating translocation events corresponding to single AMR genes. We therefore performed a multiple-sequence alignment of the class I integrons of the 5 P. aeruginosa isolates for which Nanopore sequences were generated, as the long-read data facilitate the highest-confidence assemblies. Indeed, we identified two structurally different versions of the resistance gene cassette, termed RSC1 and RSC2, the latter likely the result of multiple transposition and deletion events (Fig. 4B). Four isolates harbored wild-type cassette RSC1, while one isolate harbored RSC2. Finally, we aligned the short reads of all 49 P. aeruginosa isolates against the breakpoints distinguishing RSC1 and RSC2. We identified 21 isolates most similar to RSC1 and 9 isolates most similar to RSC2, while 10 isolates could not be uniquely assigned to one or the other, pointing to a third cassette configuration (Fig. S2). Our results indicate that AMR genes on plasmids are subject to strong selective pressure and are frequently removed, likely due to the high cost of transcribing multiple resistance genes.

### Rapid plasmid-mediated adaptation: acquisition and loss of AMR genes by horizontal gene transfer and structural rearrangement events.

**FIG 5:**
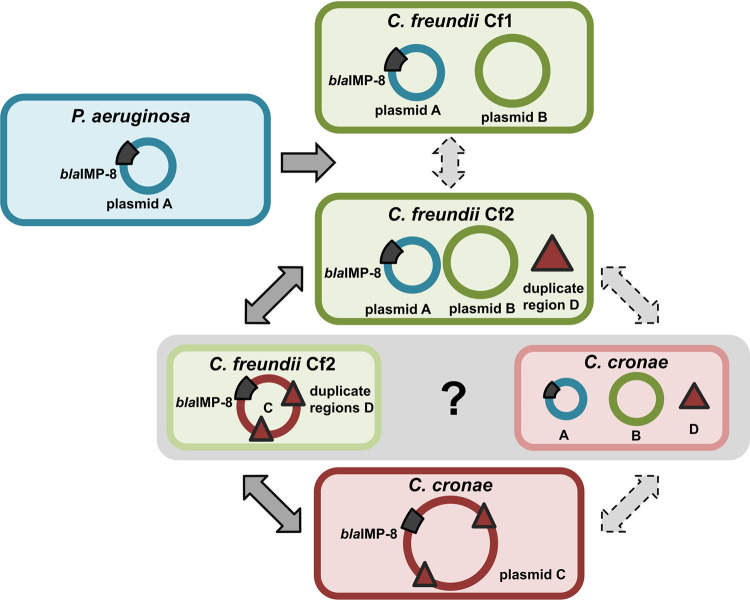
Concept of plasmid evolution and transmission across three bacterial species. P. aeruginosa *bla_I_*_MP-8_ was isolated approximately 2.5 years prior to the first isolation of C*itrobacter* species harboring *bla_I_*_MP-8_, leading to the hypothesis of a transfer of plasmid A from P. aeruginosa to *Citrobacter* species. Occurrences of the C. freundii and C. cronae *bla_I_*_MP-8_ genes started at the same time; thus, the timeline does not suggest a specific direction of the transfer. However, the existence of the transposon region in the chromosome of C. freundii cluster Cf2 (marked with a black triangle) makes it the most likely host of the merging of plasmid A and B, which are linked by two copies of the transposon region. Solid arrows represent the transmission sequence resulting from this hypothesis.

Our findings generated multiple lines of evidence indicating that the rapid gain and loss of AMR genes in opportunistic pathogens in our hospital was mediated by plasmid transfer, merging, and rearrangement, which evolved over multiple distinguishable stages (Fig. 5) in possibly the following sequence of events:

(i) Plasmid A (40 kb) harboring *bla_I_*_MP-8_ and multiple other AMR genes was transferred between P. aeruginosa and C. freundii. Although the direction of transfer cannot be determined with certainty, the fact that P. aeruginosa *bla_I_*_MP-8_ was isolated approximately 2.5 years before the first *Citrobacter bla_I_*_MP-8_ strain was detected suggests a transfer from P. aeruginosa to *Citrobacter* species. Moreover, the higher GC content of plasmid A points toward an origin of the plasmid from a background with a high level of GC content such as P. aeruginosa (average GC content of 66%). However, the possibility that an unknown intermediate host served as a reservoir for plasmid A cannot be ruled out. Following plasmid transfer to C. freundii, clonal expansion was observed; however, no clonal expansion has been seen to have occurred in C. cronae to date (Fig. 2C).

(ii) In C. freundii, the plasmid underwent further evolution resulting in the fusion of acquired plasmid A and resident plasmid B to the megaplasmid C ultimately recovered in C. cronae. We hypothesized that this happened by plasmid fusion, since plasmid C contains regions with genetic homology of close to 100% across the full length of plasmid A and plasmid B. In addition, plasmid C contained regions harboring parts of transposons which were also present in the chromosome of C. freundii cluster Cf2 strains, indicating that this organism was most likely the host of the plasmid fusion. However, the possibility of a plasmid fusion in C. cronae cannot be ruled out (Fig. 5, gray area).

(iii) We speculate that C. freundii Cf2 strains “distributed” plasmid A to C. freundii Cf1 and plasmid C to C. cronae. However, it is also possible that Cf1 and Cf2 independently acquired plasmid A from P. aeruginosa or that Cf2 acquired plasmid A from Cf1. Although less likely, the plasmid fusion resulting in plasmid C might have occurred in C. cronae after independent transfer of plasmids A and B from any of the other three bacteria. However, C. cronae is also lacking a copy of the transposon region in its chromosome which is present in cluster Cf2, making a fusion in C. cronae highly unlikely (Fig. S1). The data presented in Fig. 5 depict all possible trajectories of the adaptation processes mediated by plasmid HGT leading to three bacterial species and four clones with multiple antibiotic resistances in a single hospital within a few years.

(iv) In parallel, the class 1 integron in P. aeruginosa harboring the antimicrobial resistance genes, including *bla_I_*_MP-8_, underwent various rearrangements such as deletions and integration of AMR genes. In 12 of the P. aeruginosa isolates, one or more AMR genes were lost (Table S4), and at least 9 strains show evidence of gene cassette shuffling (Fig. S2).

In conclusion, we demonstrated the successful application of Nanopore sequencing to track the transmission and rapid evolution of an antibiotic resistance plasmid(s) within and between multiple bacterial species in a comprehensive and systematic collection of multidrug-resistant Gram-negative bacteria obtained from a large cohort of high-risk patients and corresponding environment samples.

## DISCUSSION

Understanding the evolution and spread of multidrug-resistant organisms has become a major challenge in the medical field, necessitating the development of novel diagnostic methods in order to effectively combat increasing numbers of infections with these organisms. The clinical importance of an antimicrobial resistance gene is determined by (i) the class of antibiotics that is rendered resistant, (ii) the pathogenicity of the bacterium, and (iii) the genetic location of the AMR gene. The localization of one or more AMR genes on a mobile genetic element, e.g., a plasmid, strongly increases the risk of resistance spreading between different bacterial genera, including well-adapted and successful human pathogens established in the hospital environment.

In several studies, the spread of carbapenemase gene-harboring plasmids has been demonstrated to happen in the hospital environment (see, e.g., reference 10). For example, Conlan et al. examined plasmids harboring *bla*_KPC-2_ and *bla*_KPC-3_ and provided evidence for horizontal gene transfer between Klebsiella pneumoniae, *Enterobacter* sp., and *Citrobacter* sp. (10). Interestingly, the *Citrobacter* strain described in their study (CFNIH1), which was isolated from the hospital environment, contained a 272-kb KPC-encoding plasmid and clustered very closely (core SNP distance of 28 nucleotides) with our study’s C. cronae P28 isolate (Fig. 2C), which harbored the large plasmid C. This might indicate that the genetic background of this *Citrobacter* strain enables large plasmid uptake or formation of megaplasmids in this species. The formation of megaplasmids conferring multidrug resistance has also been noted in other *Enterobacterales*. For example, Desmet et al. analyzed two clinical isolates (a Klebsiella pneumoniae strain and an Enterobacter cloacae complex strain) harboring *bla*_OXA-427_ carbapenemase and identified a 321-kb megaplasmid which resulted from a cointegration of the MDR plasmid in another plasmid background (24). A further study demonstrated that a fusion plasmid had occurred as a result of recombination in a clinical Escherichia coli isolate containing the *bla*_NDM-5_ carbapenemase gene (25). Similarly to the results seen with the C plasmid that we isolated from C. cronae, this megaplasmid also harbored duplicated transposon-containing regions, likely as a result of the fusion event. However, the fusion plasmid was not stable when transferred to an E. coli recipient strain (25). This is in line with our observations. Fusion plasmid C contained a duplicated region, most likely as a result of recombination. While plasmid C was stable within our study isolates, it was never detected afterwards, suggesting that the plasmid was not positively selected in the hospital environment. However, further studies are needed to elucidate the factors involved in megaplasmid evolution dynamics.

Although the importance of plasmid evolution and horizontal gene transfer for the spread of MDR bacteria has clearly been documented, the epidemiological surveillance of HGT within hospitals is not commonly performed on a routine basis and remains limited to few centers. Short-read sequencing technology, which is available in many hospitals, cannot reliably distinguish between plasmids and chromosomes and such analyses often lead to the occurrence of fragmented genome and plasmid assemblies. Long-read sequencing technologies, on the other hand, enable high-quality, finished assemblies of plasmids. With the emergence of Nanopore sequencing, a fast and inexpensive alternative technology for *de novo* assembly of multidrug-resistant bacteria isolates became available (11, 13, 26). Here, we demonstrated that the application of Nanopore sequencing in combination with Illumina short reads and epidemiological data enabled detailed tracking of plasmid evolution in a comprehensive consecutive collection of *bla*_IMP-8_-harboring multidrug-resistant Gram-negative bacteria. In addition to multiple plasmid-based horizontal gene transfers, we were able to detect rearrangements within the multidrug resistance gene cassette, as well as fusion of two plasmids to a megaplasmid. While the presence and absence of antimicrobial resistance genes can be postulated based on Illumina short-read assemblies, identification of their locations on mobile elements and determination of the structure of multidrug resistance gene cassettes remain challenging due to difficulties with assembling repetitive regions. In the P. aeruginosa genomes assembled using Nanopore data, we were readily able to detect continuous reads confirming the circularity of the plasmid and the exact order of the resistance gene cassette and were able to distinguish between the bacteria harboring the megaplasmid and those harboring the two independent plasmids, further emphasizing the power of long reads for determination of structures of mobile genetic elements.

### Conclusion.

The application of Nanopore sequencing and the establishment of a computational pipeline for genome and plasmid assembly, annotation, and comparative analysis (termed “*pathoLogic*,” including the novel plasmid analysis method *plasmIDent*) enabled us to investigate plasmid-driven adaptation and emergence of multidrug-resistant bacteria using a comprehensive strain collection that included patient and environment isolates. Using Nanopore-based *de novo* assemblies, we demonstrated that horizontal gene transfer between P. aeruginosa, C. freundii, and C. cronae via a multidrug resistance plasmid (plasmid fusion), resulting in a megaplasmid and evolution of the multidrug resistance gene cassette, had occurred within the short period of 3 years within our hospital. The chosen method for tracking of MDR plasmids and their evolutionary dynamics represents a powerful approach which could be applied for real-time infection control surveillance, thereby contributing to successful countermeasures and efficient containment of hospital outbreaks. In summary, we developed and showcased a novel pipeline for *de novo* bacterial genome assembly, AMR gene and plasmid characterization, and comparative analysis across species, enabling rapid tracking of AMR transmission via plasmids in hospital settings.

## MATERIALS AND METHODS

### Study isolates.

In total, 54 hospital strains were included in the study, comprising P. aeruginosa (*n* = 45), C. freundii (*n* = 8), and C. cronae (*n* = 1) strains. The strains were obtained from patient specimens, including rectal screening culture sources (*n* = 40) and water-related environment sources (toilet or sink; *n* = 14). All isolates were cultured and identified following standard microbiology protocols as described before (27) and were positive for the *bla*_IMP-8_ gene as determined by PCR (28). All isolates were recovered from samples processed in the hemato-oncology department between July 2009 and July 2015. During this time, the sampling strategy for screening cultures and environmental surveillance was adjusted as a consequence of the P. aeruginosa *bla*_IMP-8_ outbreak. Between July 2009 and October 2010, only clinical specimens were obtained. Weekly rectal screening programs of all hemato-oncology patients and environment screening of toilets, sinks, and showers in a 14-day cycle were introduced in October 2010.

### Nanopore and Illumina sequencing.

Nanopore sequencing was performed on an Oxford Nanopore Technologies MinION device with three different chemistries (versions 6, 7, and 8) and flow cell versions (FLO-MAP103 version Pk.1, FLO-MIN105 version R9, and FLO-MIN106 version R9.4). An overview of the chemistry and flow cell versions used for each sample is shown in Table S2 in the supplemental material.

### (i) ONT chemistry version 6.

Sequencing libraries were prepared with a Genomic DNA Sequencing SQK–MAP006 kit using 1.5 μg of genomic DNA (gDNA) as starting material. Briefly, nick-repaired DNA (NEBNext FFPE DNA Repair Mix; NEB) was sheared in a Covaris g-TUBE (Covaris, Inc.), followed by end repair and dA tailing (NEBNext UltraII End Repair/dA-tailing module; NEB). The leader and hairpin sequencing adapters (ONT) were ligated using blunt TA ligase (NEB). After tether addition, the final library was purified with MyOne streptavidin C1 beads (Thermo Fisher). The MinION flow cell (FLO-MAP103, ONT) was primed and loaded with the library for a 48-h run with 24-h intervals for adding new presequencing mix, running buffer, and Fuel Mix (ONT).

### (ii) ONT chemistry version 7 and 8.

Libraries were prepared with Genomic DNA Sequencing Kit SQK_NSK007 and SQK-LSK108, starting with 1.5 μg of gDNA sheared in a Covaris g-TUBE (Covaris, Inc.) and nick-repaired with NEBNext FFPE DNA repair mix (NEB). Subsequently, DNA was end-repaired and adenylated (NEBNext Ultra II End-Repair/dA-tailing module, NEB) followed by ligation of adaptor (ONT) using NEB Blunt/TA master mix (NEB). After priming of the flow cells, FLO-MIN105 libraries for kit SQK_NSK007 and FLO-MIN106 libraries for kit SQK-LSK108 were loaded and run for 48 h following the protocols of the manufacturer (ONT).

### (iii) Illumina sequencing.

Due to the advances in sequencing technology that became available over the study period, different protocols were used to obtain short-read sequences, as described before (17, 27, 29). In brief, early isolates were sequenced using 2 × 50 bp on an Illumina HiSeq 2000 sequencer (17) or using 2 × 300 bp on an Illumina MiSeq sequencer (29) or using 2 × 250 bp on a Illumina MiSeq sequencer (27). Table S2 provides a detailed overview of the sequencing protocols applied.

### Hybrid *de novo* assembly pipeline using long and short reads.

To achieve complete *de novo* genome assemblies, we developed a custom pipeline (termed *pathoLogic*; see Fig. 1) consisting of individual steps for read preprocessing, hybrid *de novo* assembly, quality control, and generation of assembly statistics. First, long Nanopore reads are subjected to adapter trimming with *Porechop* (https://github.com/rrwick/Porechop), quality filtering with *Filtlong* (https://github.com/rrwick/Filtlong), and quality control (QC) using *Nanoplot* (30). Adapter trimming and QC for short reads is performed using *SeqPurge* (31). We benchmarked multiple assembly approaches implemented in *pathoLogic*. *Unicycler*, a hybrid assembler using short and long reads (16), produced the longest contigs at high and low read coverage and was therefore used in this study. Finally, assembly statistics are calculated and contigs shorter than 2,000 bp are removed. Application-specific parameters are documented in the published source code and configuration file. All tools are included in the provided Docker image (release v1.0) available on github (*plasmIDent*, https://github.com/imgag/plasmIDent; *pathoLogic*, https://github.com/imgag/pathoLogic).

### Phylogenetic analysis.

Assembly of the short-read Illumina data for all studied isolates was performed using *Spades* version 3.7.0 (32), followed by alignment using *ProgressiveMauve* (version 2.3.1) (33) with a locally colinear block size of 1,000 bp. Phage content was removed using *Phast* (34). The obtained alignment was used for phylogeny calculation, applying IQ tree version 1.6.3 in UFboot mode with parameters modelFinder and 1,000 bootstraps (35–37). For visualization, *Figtree* version v1.4.2 (http://tree.bio.ed.ac.uk/software/figtree/) was applied. For calculation of the *Citrobacter* maximum likelihood phylogeny, the 9 study isolates and 14 reference genomes were included as follows: Citrobacter amalonaticus Y19 (CP011132_1), Citrobacter braakii GTA-CB01 (JRHK01000001.1), C. braakii GTA-CB04 (JRHL01000001.1), Citrobacter farmeri GTC 1319 (NZ_BBMX01000031.1), C. freundii CFNIH1 (NZ_CP007557_1), Citrobacter rodentium ICC168 (NC_013716_1), Citrobacter sedlakii NBRC 105722 (NZ_BBNB01000030.1), C. werkmanii NBRC 105721 (NZ_BBMW01000009.1), Citrobacter youngae ATCC 29220 (NZ_GG730308.1), Citrobacter koseri ATCC BAA-895 (CP000822.1), C. freundii ATCC 8090 (JMTA01000001.1), C. rodentium ATCC 51459 (JXUN01000001.1), C. amalonaticus L8A (JMQQ01000001.1), and C. werkmanii DSMZ17579 (29). The P. aeruginosa phylogeny was calculated as described before (38) with minor changes (applying IQ trees as described above instead of RaXML), including the 45 study isolates and 1 P. aeruginosa *bla*_VIM-2_ outgroup strain (P_3, P. aeruginosa [27]; European Nucleotide Archive [ENA] accession number PRJEB21865).

### Plasmid detection and annotation.

For most isolates, the assembly produced one or a few large chromosomal scaffolds along with several shorter contigs (between 10 kb and 200 kb in length). The latter might have stemmed either from complete circular plasmids or from fragments of the chromosome or plasmids. We therefore developed the *plasmIDent* tool, which uses long reads to ascertain whether a scaffold is circular, identifies all antibiotic resistance genes, and calculates characteristic metrics such as GC content and read coverage. *PlasmIDent* takes assembled genomes in fasta format and Nanopore reads in fastq format as input. First, contig ends are fused in order to mimic a circular layout. Next, *minimap2* is used to align Nanopore reads to the putative plasmid and the end-to-end fusion site. In cases in which long reads continuously cover the scaffold and the artificially closed gap, we assume that the sequence originated from a circular plasmid. Furthermore, sudden changes of median GC content within the plasmid are used to predict ancestral fusions of two or multiple plasmids. Finally, *plasmIDent* supports discovery of resistance genes using the CARD database.

### Genome annotations.

Assembled FASTA files were uploaded to the *ResFinder* tool (https://cge.cbs.dtu.dk//services/ResFinder/), applying a 98% identity threshold and a minimum overlapping length of 60%. The P. aeruginosa sequence type was extracted using *ResFinder*. Additionally, CARD-based annotations automatically generated by *plasmIDent* were merged with the *ResFinder* results. Finally, we used the *RAST* Web server to obtain complete genome and plasmid annotations for all isolates and the *ISFinder* Web server to specifically identify transposons and insertion sequences. We displayed the best hits and annotated the transposons or *IS* elements to the family level in the duplicated regions.

### Comparative genome and plasmid analysis across species. (i) Whole-genome alignment (WGA).

Multiple whole-genome alignments of all assembled plasmids were generated with *progressiveMauve* in order to find highly similar regions. Plasmids with highly homologous regions were additionally compared by pairwise sequence alignment using nucmer (see, e.g., Fig. S1 in the supplemental material), resulting in a pairwise identity score and the annotation of homologous regions. We used dot plots (*pathoLogic* utility scripts) of the pairwise alignments to visually identify rearrangements in plasmids. Homologous regions between plasmids and chromosomal scaffolds were identified using pairwise alignment (nucmer) between a plasmid of interest and the concatenated sequence representing all scaffolds in an isolate’s genome assembly. More specifically, we identified homologous sequences of the transposon-containing region found in plasmid C but not in plasmid A and plasmid B in order to ascertain whether a *Citrobacter* isolate contained only plasmids A and B and the transposon-containing region inserted in the chromosome or contained plasmid C with the transposon-containing region in the plasmid.

### (ii) Read coverage (density) analysis.

We chose megaplasmid C of isolate 28_P_CC as the reference plasmid, as it integrates both plasmid A and plasmid B involved in the studied horizontal gene transfer of AMR genes. We used bwa-mem to realign Illumina short reads of each isolate to the reference plasmid, thereby determining the presence or absence of specific regions based on read density (i.e., whether regions without read coverage were absent in a studied isolate; see Fig. 4 [see also Fig. S2]). We identified breakpoints, indicating structural variants or the end of plasmids, based on clip or split reads. We defined deletions as regions with very-low-density read coverage, with split or paired reads spanning the two breakpoints. (Plasmid ends were identified by circularization as described before.) Furthermore, we evaluated whether putatively deleted resistance genes were also absent from the plasmid AMR gene annotations by ResFinder and CARD.

### (iii) AMR gene rearrangements.

WGA of the resistance gene cassette of all isolates assembled with Nanopore reads identified two haplotypes, termed RSC1 and RSC2, distinguished by two translocations of AMR genes. In order to assign all sequenced isolates to one or the other cassette configuration, we aligned Illumina short reads to the 4 breakpoints per haplotype (two breakpoints for each translocation event per cassette configuration). Then, we compared the numbers of aligned reads spanning the four breakpoints in RSC1 versus RSC2 and computed the log-transformed fraction of breaks in RSC1 and RSC2, each normalized by the corresponding amount of total reads. Isolates showing log values above 1 were assigned to RSC1 and those showing log values below −1 to RSC2, while the other isolates remained unassigned.

### Data availability.

All sequence data have been deposited at the European Nucleotide Archive (study accession number PRJEB31907). *pathoLogic* (https://github.com/imgag/pathoLogic) and *plasmIDent* (https://github.com/imgag/plasmIDent) were developed in this study and are freely available on GitHub.
