# Supplementary material for: Tracking of Antibiotic Resistance Transfer and Rapid Plasmid Evolution in a Hospital Setting by Nanopore Sequencing
Source: mSphere. 2020 Aug 19;5(4):e00525-20. doi: 10.1128/mSphere.00525-20 (PMC7440845; doi:10.1128/mSphere.00525-20)
Supplement: TABLE S1 [file mSphere.00525-20-st001.pdf]

Sample	Number of reads:	Median read length:	Median read quality:	Flowcell version:	ONT Kit:
28_P_CC	107501	3731	7.7	FLO-MAP103	SQK_MAP006
30_P_CF	449375	3300	9.4	FLO-MIN106	SQK-LSK108
32_P_CF	689747	3134	9	FLO-MIN106	SQK-LSK108
34_P_CF	640898	3163	9.8	FLO-MIN106	SQK-LSK108
9_E_CF	59275	1572	7.8	FLO-MIN106	SQK-LSK108
38_P_CF	327411	5908	8.6	FLO-MIN106	SQK-LSK108
27_P_CF	89853	1918	6.9	FLO-MIN105	SQK-NSK007
29_P_CF	926772	2753	9.7	FLO-MIN106	SQK-LSK108
13_E_CF	224377	2136	9.1	FLO-MIN106	SQK-LSK108
9_P_PA	71923	4373	8.5	FLO-MIN105	SQK-NSK007
11_P_PA	50986	6222	8.8	FLO-MIN105	SQK-NSK007
37_P_PA	137180	3542	6.5	FLO-MIN105	SQK-NSK007
39_P_PA	41934	1340	3.9	FLO-MIN105	SQK-NSK007
23_P_PA	149047	2127	6.6	FLO-MIN106	SQK-LSK108
