# Supplementary material for: Tracking of Antibiotic Resistance Transfer and Rapid Plasmid Evolution in a Hospital Setting by Nanopore Sequencing
Source: mSphere. 2020 Aug 19;5(4):e00525-20. doi: 10.1128/mSphere.00525-20 (PMC7440845; doi:10.1128/mSphere.00525-20)
Supplement: TABLE S2 [file mSphere.00525-20-st002.pdf]

Sample ID	# contigs	Largest contig	Total length	GC (%)	N50	N75	L50	L75
28_P_CC	3	5062321	5230854	52.09	5062321	5062321	1	1
30_P_CF	6	4745085	5272837	51.88	4745085	4745085	1	1
32_P_CF	5	5038088	5260813	51.87	5038088	5038088	1	1
34_P_CF	5	4892588	5110715	51.71	4892588	4892588	1	1
9_E_CF	29	1444960	5262884	51.80	636697	296195	3	6
38_P_CF	6	4889818	5112060	51.69	4889818	4889818	1	1
27_P_CF	25	1834568	5388324	51.84	915012	298121	2	5
29_P_CF	10	2415109	5401457	51.80	2241041	2241041	2	2
13_E_CF	11	3937886	5105413	51.69	3937886	3937886	1	1
9_P_PA	18	1941814	6786480	66.14	1894580	572642	2	4
11_P_PA	14	1894702	6808107	66.12	1693847	948850	2	4
37_P_PA	20	1907170	6818725	66.11	1047546	547243	3	5
39_P_PA	19	1988153	6650226	66.21	1549117	1486086	2	3
23_P_PA	36	1047588	6766190	66.14	466918	315712	5	9
