# Supplementary material for: Tracking of Antibiotic Resistance Transfer and Rapid Plasmid Evolution in a Hospital Setting by Nanopore Sequencing
Source: mSphere. 2020 Aug 19;5(4):e00525-20. doi: 10.1128/mSphere.00525-20 (PMC7440845; doi:10.1128/mSphere.00525-20)
Supplement: TABLE S3 [file mSphere.00525-20-st003.pdf]

A)

Strain Id	Species	# Plasmids	Plasmid types
9_P_PA	*P. aeruginosa*	4	A , E, F
11_P_PA	*P. aeruginosa*	3	A, E
37_P_PA	*P. aeruginosa*	3	A, E
39_P_PA	*P. aeruginosa*	3	A', E
28_P_CC	*C. cronae*	2	C
30_P_CF	*C. freundii*	3	A, B
32_P_CF	*C. freundii*	4	A, B, D
34_P_CF	*C. freundii*	4	A, F, B',G,
9_E_CF	*C. freundii*	3	A, B
38_P_CF	*C. freundii*	5	A, B'', G', H
23_P_PA	*P. aeruginosa*	3	A, E
27_P_CF	*C. freundii*	4	A, B, I
29_P_CF	*C. freundii*	4	A, B, J
13_E_CF	*C. freundii*	5	A, B'', G', H

B)

Plasmid	Length	GC %	Res. Genes	Closest Hit (NCBI)	Query cover	Identity %
A	39882	58,92	yes	LT837805.1	63%	97
A'	39371	58,82	yes	LT837805.2	64%	97
B	88318	45,02	no	CP033074.1	39%	78
B'	86220	45,09	no	CP033074.2	37%	78
B''	85137	45,18	no	CP033074.2	38%	78
C	163147	51,8	yes	CP011977.1	18%	99
D	89139	52,09	no	CP024881.1	98%	99
E	7173	56,6	no	CP027172.1	94%	99
F	2009	63,27	no	LN853727.1	14%	75
G	86638	49,21	no	CP023978.0	5%	98
G'	85406	49,24	no	CP023978.1	4%	98
H	6431	39,08	no	AY842156.1	4%	86
I	76389	51,84	no	CP016762.1	79%	99
J	162283	49,42	no	CP024882.1	73%	99
