# Supplementary figures and images for: Tracking of Antibiotic Resistance Transfer and Rapid Plasmid Evolution in a Hospital Setting by Nanopore Sequencing
Source: mSphere. 2020 Aug 19;5(4):e00525-20. doi: 10.1128/mSphere.00525-20 (PMC7440845; doi:10.1128/mSphere.00525-20)

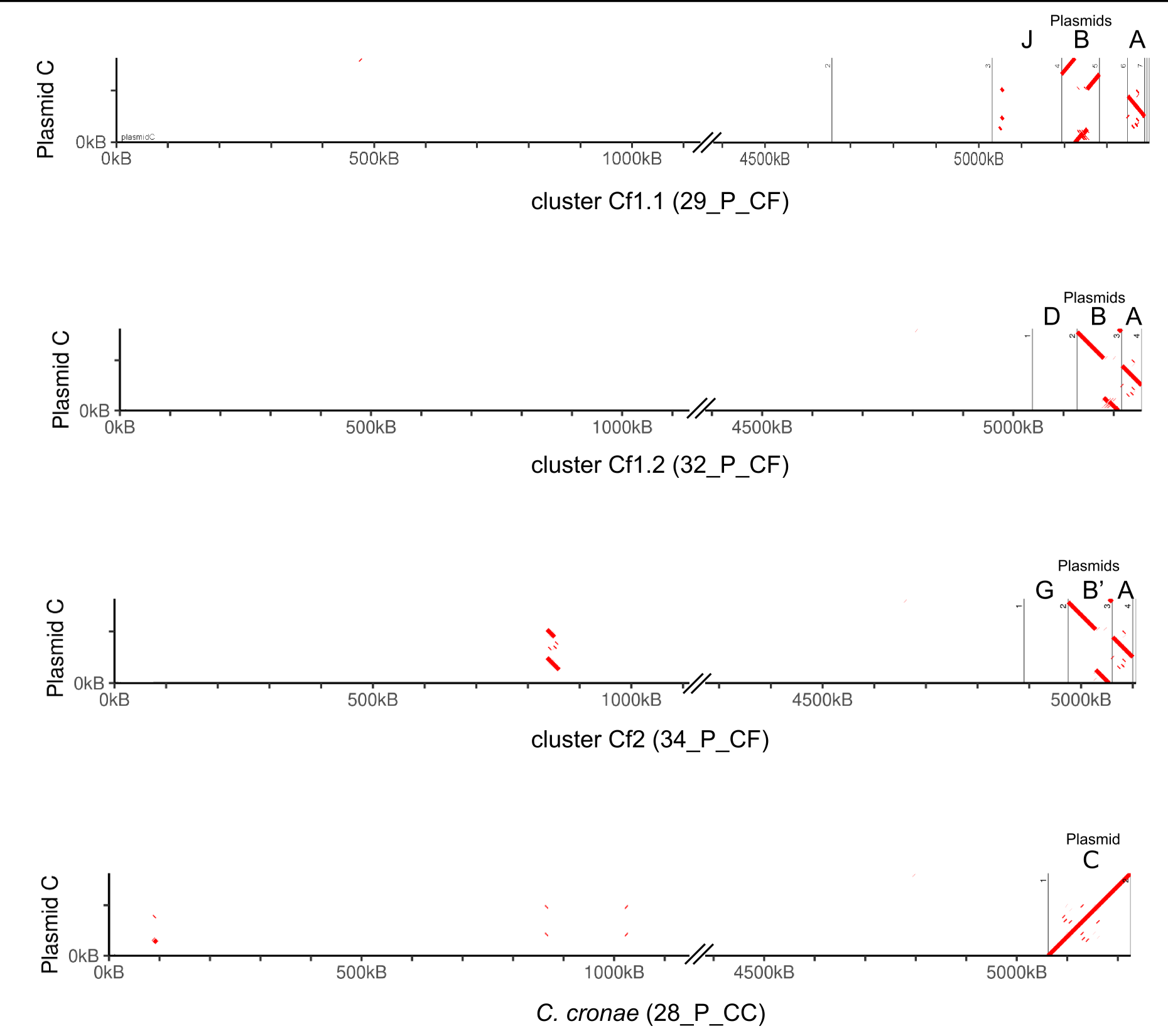

Supplement: FIG S1 [file mSphere.00525-20-sf001.tif]

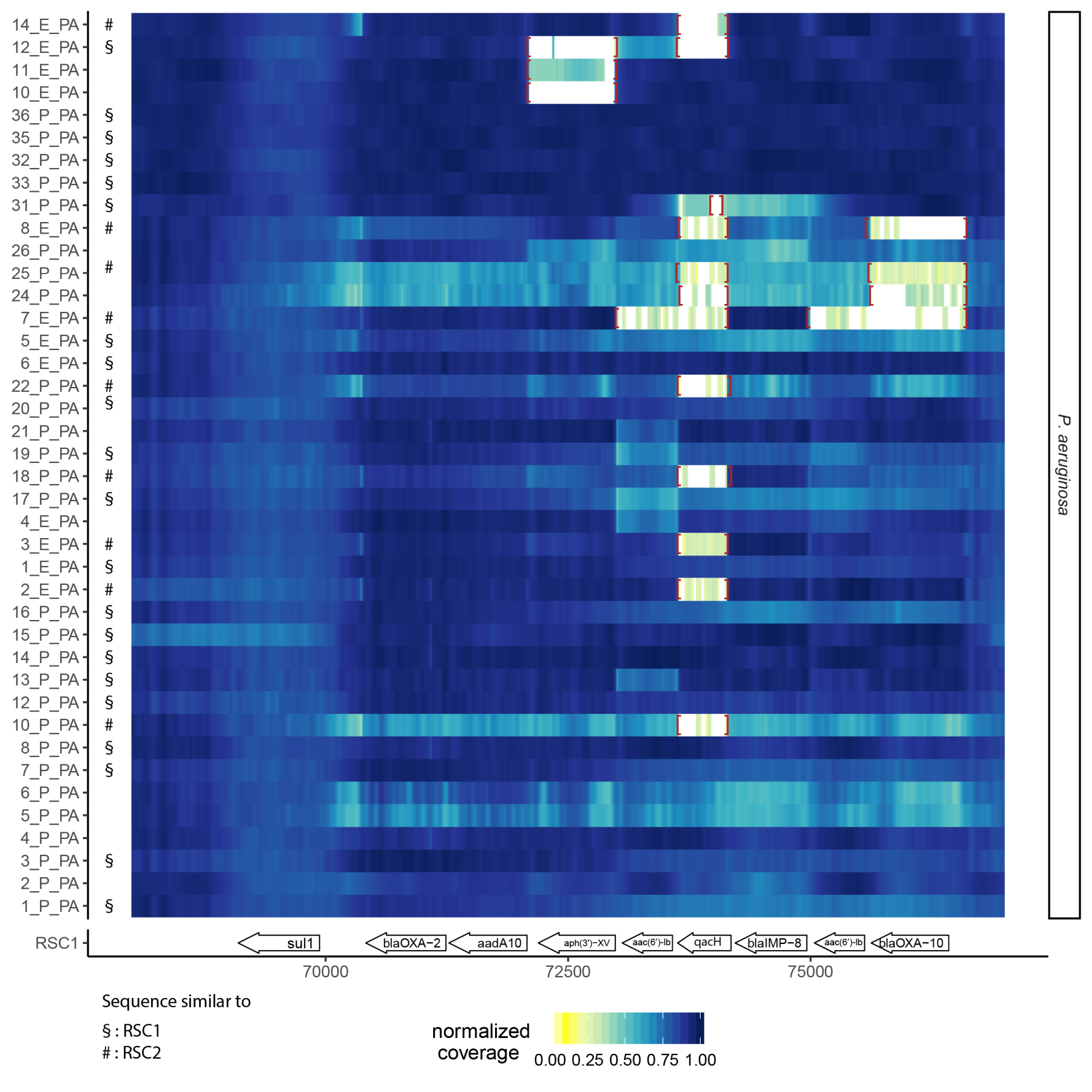

Supplement: FIG S2 [file mSphere.00525-20-sf002.tif]

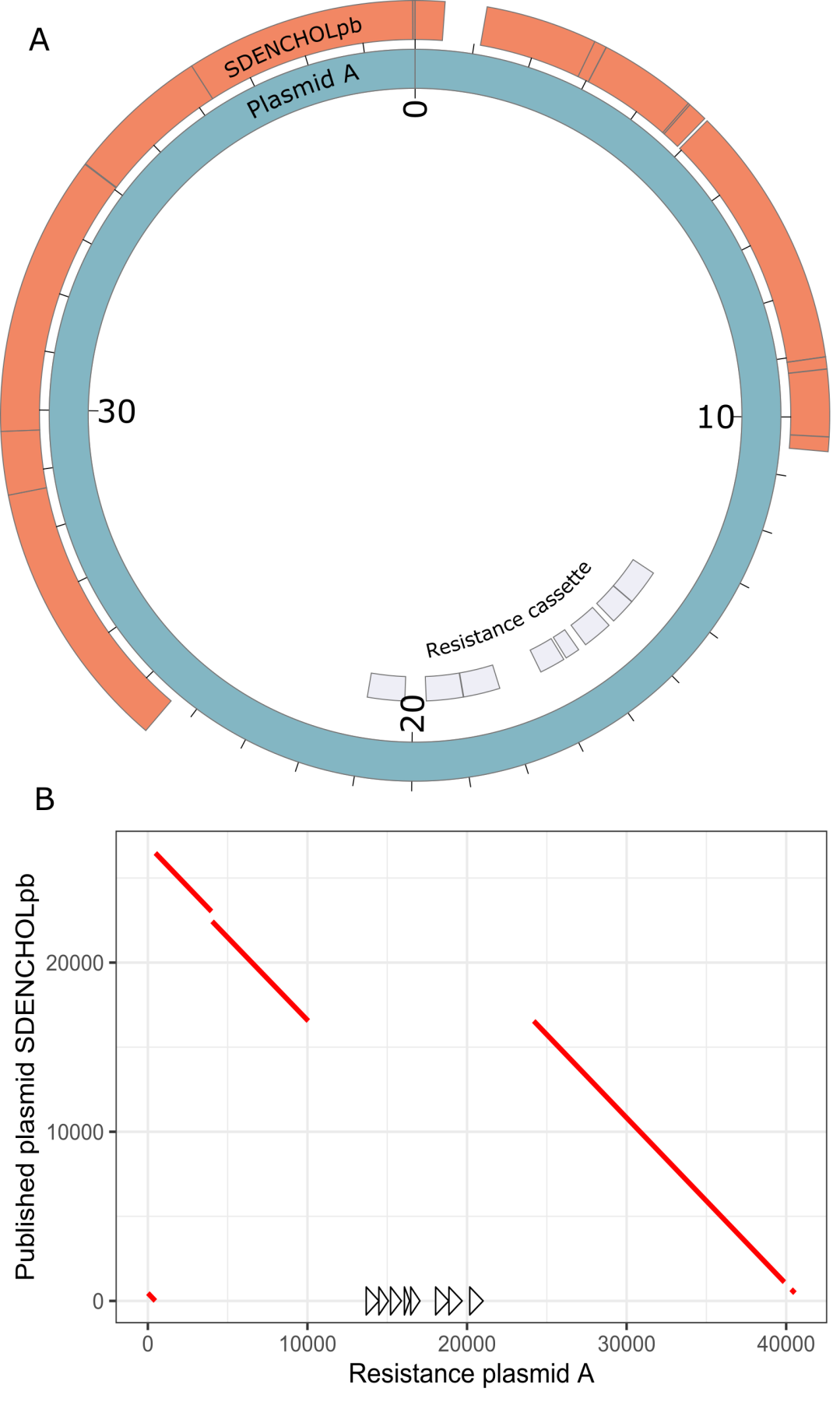

Supplement: FIG S3 [file mSphere.00525-20-sf003.tif]
