# Supplementary material for: Tracking of Antibiotic Resistance Transfer and Rapid Plasmid Evolution in a Hospital Setting by Nanopore Sequencing
Source: mSphere. 2020 Aug 19;5(4):e00525-20. doi: 10.1128/mSphere.00525-20 (PMC7440845; doi:10.1128/mSphere.00525-20)
Supplement: TABLE S4 [file mSphere.00525-20-st004.pdf]

Sample ID	Missing resistance genes
27_P_C	qacH
10_E_PA	aph(3')-XV
10_P_PA	qacH
11_E_PA	aph(3')-XV
12_E_PA	aph(3')-XV, qacH
14_E_PA	qacH
19_P_PA	qacH
22_P_PA	qacH
24_P_PA	qacH, blaOXA-10
25_P_PA	qacH, blaOXA-10
31_P_PA	qacH (partial)
7_E_PA	aac(6')-lb-cr, qacH, aac(6')-lb-cr, blaOXA-10
8_E_PA	qacH, blaOXA-10
